# Degradation of Aflatoxin B_1_ during the Fermentation of Alcoholic Beverages

**DOI:** 10.3390/toxins5071219

**Published:** 2013-06-28

**Authors:** Tomonori Inoue, Yasushi Nagatomi, Atsuo Uyama, Naoki Mochizuki

**Affiliations:** Research Laboratory for Food Safety Chemistry, Asahi Group Holdings, Ltd., 1-21, Midori 1-chome, Moriya-shi, Ibaraki 302-0106, Japan; E-Mails: tomonori.inoue@asahigroup-holdings.com (T.I.); yasushi.nagatomi@asahigroup-holdings.com (Y.N.); atsuo.uyama@asahigroup-holdings.com (A.U.)

**Keywords:** aflatoxin B_1_, beer, wine, fermentation, high pressure liquid chromatography tandem mass spectrometry (LC-MS/MS)

## Abstract

Aflatoxin B_1_ (AFB_1_) is a contaminant of grain and fruit and has one of the highest levels of carcinogenicity of any natural toxin. AFB_1_ and the fungi that produce it can also contaminate the raw materials used for beer and wine manufacture, such as corn and grapes. Therefore, brewers must ensure strict monitoring to reduce the risk of contamination. In this study, the fate of AFB_1_ during the fermentation process was investigated using laboratory-scale bottom and top beer fermentation and wine fermentation. During fermentation, cool wort beer samples and wine must samples were artificially spiked with AFB_1_ and the levels of AFB_1_ remaining after fermentation were analyzed. AFB_1_ levels were unchanged during both types of fermentation used for beer but were reduced to 30% of their initial concentration in wine. Differential analysis of the spiked and unspiked wine samples showed that the degradation compound was AFB_2a_, a hydrated derivative of AFB_1_. Thus, the results showed that the risk of AFB_1_ carryover was still present for both types of beer fermentation but was reduced in the case of wine fermentation because of hydration.

## 1. Introduction

Mycotoxins are frequent contaminants of grain and fruit. Fungi and mycotoxins are potential problems for farmers and food producers because they can adversely affect production. Close attention should therefore be paid to the risk of contamination. In our previous studies, we investigated the fate of mycotoxins in several different kinds of alcoholic beverages [1,2,3]. Such studies are required in order to understand the fate of these toxins during manufacturing, thereby improving risk management and reducing the potential for mycotoxin contamination. 

In the current study, we focused on the fate of aflatoxin B_1_ during the fermentation processes used for beer and wine. AFB_1_ has one of the highest levels of carcinogenicity of any natural toxin and has been classified as a Group 1 (carcinogenic to humans) compound by the International Agency for Research on Cancer (IARC) [4]. In a putative pathway of carcinogenicity, cytochrome P450 metabolizes ingested AFB_1_ to AFB_1_-8-9-epoxide, which then covalently reacts with DNA [5,6,7]. AFB_1_ can contaminate both beer [8,9] and raw materials, such as barley [10,11] and corn [12,13], which are used for the production of beer. AFB_1_ and *Aspergillus flavus*, a fungus that is known to produce AFB_1_, can also be found in the grapes or must (*i.e*., pressed grapes) used for wine [14,15].

In this study, we focused on beer and wine, that is, alcoholic beverages with high levels of annual global consumption. Beer can be classified into two groups on the basis of the brewing method used: top-fermented beer and bottom-fermented beer. Historically, top-fermented beer has been the traditional type of beer commonly produced in European countries. This type of beer is fermented using *Saccharomyces cerevisiae*, typically at 15–25 °C for 1 week. Bottom-fermented beer became popular only from the 19th century onwards but is now the most commonly consumed type of beer worldwide. Bottom-fermented beer is fermented using *S. pastorianus*, typically at 6–12 °C for 7–10 days (primary) and at 1–10 °C for several weeks (secondary for maturation) [16]. The fermentation conditions used for wine are similar to those used for top-fermented beer, in that *S. cerevisiae* is used at 20–30 °C for wine. 

In this study, cool wort and must were spiked with AFB_1_ and fermented in a laboratory-scale setup under three different conditions to determine the fate of AFB_1_ and help predict the degree of contamination risk.

## 2. Results and Discussion

### 2.1. Method Validation

The analysis methods for AFB_1_ were validated using cool wort (pre-fermented beer), beer, yeast crop, must, wine, and wine lees (deposits of residual yeast and other particles), that is, the interim and final products of both beer and wine. The conditions used for validation are described in Section 3.7. The ideal criteria for validation were defined as follows: relative standard deviation of repeatability (RSD) of less than 20%, recovery between 70% and 120%, and correlation coefficients for linearity (*r*) of more than 0.99. Cool wort, beer, must, wine, and wine lees all provided good results, as shown in Table 1. However, the results for the yeast crop were ideal in terms of repeatability and linearity but not in terms of recovery. Hence, in this study, a standard addition calibration curve was used to estimate residual concentrations. 

**Table 1:** Results of method validation for Aflatoxin B_1_ (AFB_1_) in each sample. The standard deviation of repeatability (RSD) was calculated at 5 ng·mL^−1^ (*n* = 5). Linearity (*r*) was estimated from eight data points, that is, 0.5, 1, 2, 5, 10, 20, 50, and 100 ng·mL^−1^, and recovery was calculated at 5 ng·mL^−1^ (*n* = 5).

	RSD (%)	Linearity (*r*)	Recovery (%)
Cool wort	5.5	1.00	87
Beer	1.7	1.00	88
Yeast crop	4.3	1.00	43
Must	5.7	1.00	91
Wine	4.4	1.00	96
Wine lees	4.5	1.00	71

### 2.2. Fate of Mycotoxins during Beer Brewing

The residual ratio of AFB_1_ during bottom fermentation with *S. pastorianus* has been calculated and published as part of our previous study [3]. These results showed that AFB_1_ concentration did not change significantly during the bottom-fermentation process. After fermentation, approximately 5% of the initial contaminant AFB_1_ was found on the yeast surface. This finding indicated that adsorption upon yeast settling was the main reason for a drop in AFB_1_ concentrations from 100% to 82% during fermentation. 

**Figure 1:**
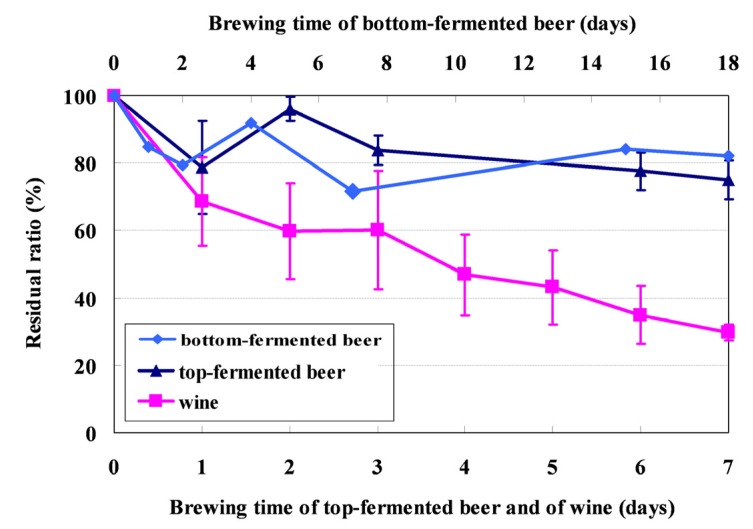
Residual ratios of AFB_1_ during fermentation. Each residual ratio has been plotted with the standard deviation (SD) bar of four replicate experiments. The residual ratio of bottom-fermented beer was plotted from one experiment without SD bar.

Top fermentation is an alternative method used for brewing beer. It differs from bottom fermentation in the type of yeast strain, fermentation temperature, and fermentation period used, as indicated in Section 3.4.1, “Laboratory-Scale Beer Fermentation”. We investigated the fate of AFB_1_ by performing top-beer fermentation trials for 7 d in the presence of *S. cerevisiae* in four replicate experiments. The residual ratios of AFB_1_ present were calculated from cool wort artificially spiked with AFB_1_. By the 7th day, the residual concentration of AFB_1_ had decreased to 75.1% of the initial level (Figure 1). Interestingly, although the AFB_1_ level decreased to 78.8% of its initial concentration on the first day, it increased to 96.0% again on the second day. This finding suggests that AFB_1_ was taken up by the foam produced during the early phase of fermentation and that, after the foam disappeared, AFB_1_ was released again into the wort. The adsorption ratio for the yeast crop was also analyzed after the fermentation process was complete. In contrast with the results for bottom fermentation, AFB_1_ was not adsorbed on the yeast surface.

### 2.3. Fate of Aflatoxins during Vinification

The fermentation conditions examined for wine were similar to those used for top-fermented beer, where the temperature was 25 °C, the yeast strain used was *S. cerevisiae*, and the length of the fermentation period was 7 days. The residual ratio in the fermenting must was analyzed every day in 4 replicate experiments. The results are shown in Figure 1. During the fermentation process, the AFB_1_ levels gradually decreased to 29.6% of the initial concentration (Figure 1). Wine lees were collected by centrifugation after fermentation, and the adsorption ratio was analyzed. The results showed that 7.2% of the AFB_1_ present was adsorbed on the wine lees. Hence, approximately 60% of the spiked AFB_1_ had been converted into other compounds. 

### 2.4. Identification of Degradation Compounds

**Figure 2:**
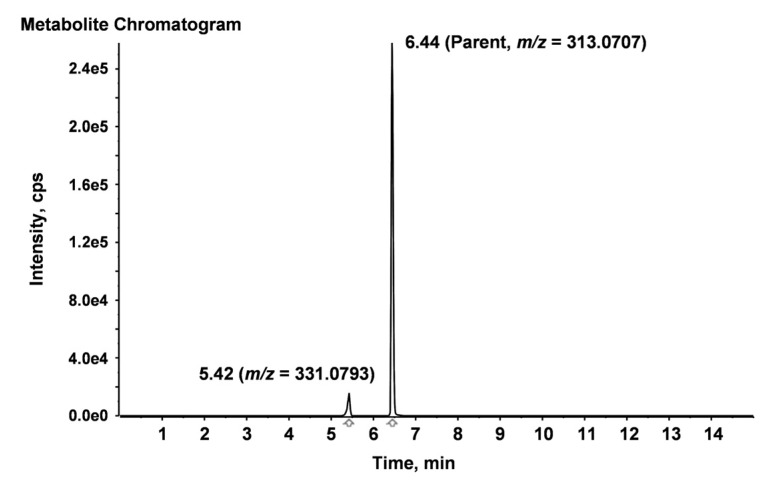
Result of a differential analysis: AFB_1_-related peaks for fermented wine prepared from must spiked with AFB_1_.

**Figure 3:**
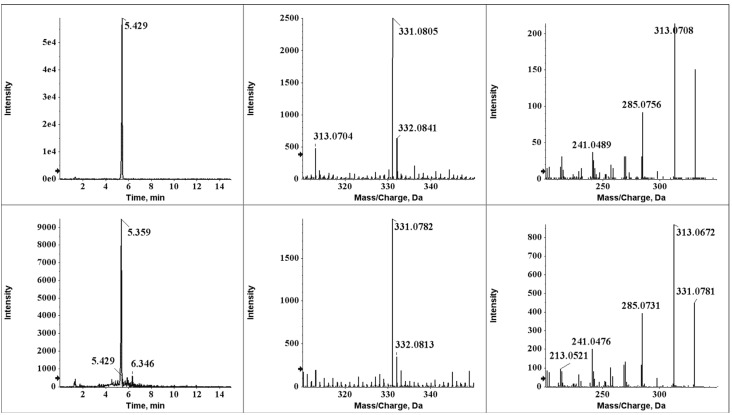
XIC chromatogram (left), MS spectra (center), and MS/MS spectra (right) of the degradation compound (upper) and synthesized AFB_2a_ (bottom).

**Figure 4:**
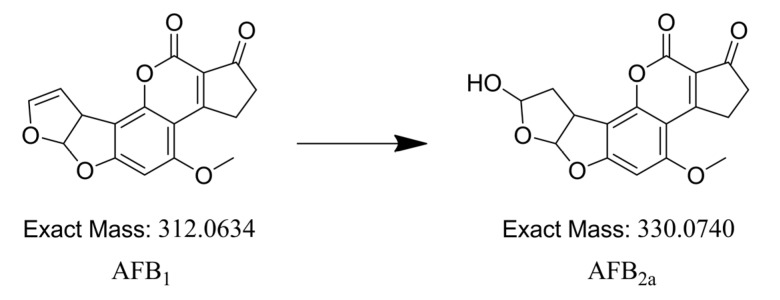
Conversion of AFB_1_ to AFB_2a_: Structure and exact mass.

In order to determine the safety of wine for consumption, it is important that the degradation compounds of AFB_1_ be identified. Therefore, liquid chromatography-interfaced quadrupole time-of-flight hybrid tandem mass spectrometry (LC-q-TOFMS) was used. Because q-TOFMS provided high resolution (mearurement at the milli-mass unit level), it could be used to separate the compound peaks of the degradation products from those of all the other wine components. Moreover, this approach narrowed down the range of possible candidates in terms of their composition formula and facilitated the estimation of structure. The actual degradation compounds were determined using differential analysis of fermented must samples spiked or unspiked with AFB_1_. These samples were analyzed in the full-scan mode by using q-TOFMS. Analysis of the differences in the peaks for AFB_1_-spiked and unspiked samples with Metabolite Pilot (AB Sciex, Foster City, CA) helped identify a new peak, implicated as a relevant compound, at 5.4 min (Figure 2). This compound had a mass-to-charge ratio (*m/z*) of 331.0793, whereas AFB_1_, the original compound, had an *m*/*z* of 313.0707. This result indicated that the new compound had been modified with an *m/z* of 18.0086 to AFB_1_ and that one of the candidates was hydrated AFB_1_, assuming that the new compound was detected as proton-adducted ions. This hypothesis was supported by the finding that the new compound was detected earlier than AFB_1_ on the ODS column. Collectively, these results suggested that the new compound had a much more polar structure than AFB_1_, which could be due to hydration. Here, AFB_2a_ is known to be a hydrolyte of AFB_1_, and it has been successfully synthesized chemically [17]. Therefore, each fermented sample contaminated with AFB_1_ and chemically synthesized AFB_2a_ was analyzed by LC-q-TOFMS. The data acquired for the new compound and AFB_2a_ showed homology in terms of retention time, *m/z* at high resolution, and the fragmentation pattern of ms/ms spectra (Figure 3). Therefore, we concluded that AFB_1_ was converted to AFB_2a_ which is the less toxic compound [18,19], during the process of wine fermentation (Figure 4). 

### 2.5. Aflatoxin B_2a_ during Beer Fermentation

It was found that AFB_1_ was converted to AFB_2a_ during vinification. Although the AFB_1_ levels did not decrease substantially during beer fermentation, AFB_2a_ production was confirmed by high-sensitivity tandem mass spectra in the multiple reaction monitoring (MRM) mode. AFB_2a_ was never detected during the bottom fermentation of beer. However, trace levels of AFB_2a_ were detected in top-fermented beer. In conclusion, although both vinification and brewing are based on fermentation carried out by yeast, AFB_1_ appears to be preferentially hydrated during the wine-making process only. This finding may be explained by differences with respect to the yeast, fermentation conditions, or raw materials used. Specifically, although *S. cerevisiae* was used in both the processes, different strains were present in wine versus beer fermentation. Furthermore, the fermentation conditions for wine (25 °C and pH 3) were distinct from those for beer (20 °C and a change in pH from 5 to 4). In addition, the raw materials used for wine making (grapes) are different to those used to brew beer (wort made from barley, corn, and hops).

## 3. Experimental Section

### 3.1. Materials

AFB_1_ standard solutions (3 μg·mL^−1^ in benzene:acetonitrile = 98:2 solution, and 20 μg·mL^−1^ in an acetonitrile solution) were purchased from Supelco, Inc. (Bellefonte, PA, USA). Acetonitrile (LC/MS grade), methanol (LC/MS grade), and ammonium acetate (JIS-guaranteed reagent), purchased from Kanto Chemical Co., Inc. (Tokyo, Japan), and acetic acid (LC/MS grade), obtained from Wako Pure Chemical Industries, Ltd. (Osaka, Japan), were used as solvents. Sample preparation was conducted using an Oasis HLB cartridge (6 cc, 200 mg) provided by Waters (Milford, MA, USA) and a MultiSep #229 Ochra solid phase cartridge (Romer, Union, MO, USA). Analytical samples were filtered using a PTFE filter with a mesh size of 0.2 μm, purchased from Advantec Toyo Kaisha, Ltd. (Tokyo, Japan). Dried top-fermentation yeast for beer was purchased from Fermentis (Marcq-en-Baroeul, France), while dried wine yeast was purchased from Shinwa Foods Chemical Co. Ltd. (Tokyo, Japan). 

### 3.2. Apparatus

The samples were analyzed using a liquid chromatography-tandem mass spectrometry (LC-MS/MS) system. A highly sensitive tandem mass spectrometry system, comprising a Xevo TQ MS system (Waters, Milford, MA, USA) interfaced with an Acquity UPLC system (Waters) equipped with an Acquity BEH C_18_ column (1.7 μm, 2.1 × 50 mm), was used. A high-resolution tandem mass spectrometry system (LC-qTOF), comprising a triple TOF 5600 system (AB Sciex, Foster City, CA, USA) and a Nexera UHPLC system (Shimadzu, Kyoto, Japan) equipped with an Acquity BEH C18 column (1.7 μm, 2.1 × 100 mm), was also used.

### 3.3. Analytical Method

#### 3.3.1. High-Sensitivity Tandem Mass Spectrometry

The concentration of each sample was analyzed with high-sensitivity tandem mass spectrometry under the following analytical conditions: Five microliters of each sample was injected into the column with the temperature controlled at 40 °C and the flow rate of gradient elution set at 0.5 mL·min^−1^. The mobile phase solvents consisted of 2 types of eluents: (a) water and (b) 2% acetic acid and 0.1 mM ammonium acetate in methanol.

Analysis was performed under the following conditions: 0 min (95% A, 5% B), 4.5 min (20% A, 80% B), 4.51 min (95% A, 5% B), and 6 min (95% A, 5% B). In order to avoid matrix effects, the following conditions were used for yeast: 0 min (95% A, 5% B), 10 min (20% A, 80% B), 10.5 min (95% A, 5% B), and 11 min (95% A, 5% B). Target substances were ionized in the positive electrospray ionization (ESI) mode. The MS/MS properties were set as follows: ion source voltage, 3 kV; ion source temperature, 150 °C; and desolvation temperature, 500 °C. Data for quantification and confirmation were acquired in the MRM mode. The precursor-to-fragment transitions (optimum cone and collision energies) of AFB_1_ were 313.1 to 240.9 (40 V and 35 V, respectively) for quantification and 313.1 to 268.7 (40 V and 32 V, respectively) for confirmation and of AFB_2a_ were 331.3 to 313.1 (34 V and 18 V, respectively) for quantification and 331.3 to 268.7 (34 V and 24 V, respectively) for confirmation.

#### 3.3.2. High-Resolution Tandem Mass Spectrometry

The degradation compound was assigned using high-resolution tandem mass spectrometry under the following analytical conditions: Five microliters of each sample was injected into the column with the temperature controlled at 40 °C and the flow rate of gradient elution set at 0.2 mL·min^−1^. The mobile phase solvents consisted of 2 types of eluents: (a) 5 mM ammonium acetate in water and (b) acetonitrile.

Analysis was conducted under the following conditions: 0 min (95% A, 5% B), 1 min (95% A, 5% B), 9 min (0% A, 100% B), 10 min (0% A, 100% B), 10.1 min (95% A, 5% B), and 15 min (95% A, 5% B). The target substances were ionized in the ESI mode. The MS properties were set as follows: scan range, *m*/*z* of 100–1000; accumulation time, 0.1 s; ion source gas 1, 50 psi; ion source gas 2, 50 psi; curtain gas, 25 psi; desolvation temperature, 500 °C; ion spray voltage floating, 5500 V; declustering potential, 80 V; and collision energy, 10 V. MS/MS data were acquired in the information-dependent acquisition (IDA) mode. The MS/MS properties were set as follows: scan range, *m*/*z* of 40–1000; accumulation time, 0.025 s; ion source gas 1, 50 psi; ion source gas 2, 50 psi; curtain gas, 25 psi; desolvation temperature, 500 °C; ion spray voltage floating, 5500 V; declustering potential, 80 V; collision potential, 35 V; and collision energy spread, 15 V.

### 3.4. Laboratory-Scale Fermentation

#### 3.4.1. Laboratory-Scale Beer Fermentation

Bottom fermentation was performed in our previous study on mycotoxin fate [3]. Briefly, cool wort spiked with mycotoxins was added to yeast (*S. pastorianus*, 1 g), and fermentation was performed at 10 °C for the first 7 days, using a magnetic stirrer. Further fermentation was performed under static conditions at 10 °C for the next 7 days and then at 4 °C for the last 4 days. 

For top-fermented beer, cool wort was prepared using a process described in our previous study [2,3]. The noncontaminated cool wort (200 mL) was spiked with AFB_1_ standard solution at a concentration of 10 ng·mL^−1^. In top-beer fermentation trials, yeast (*S. cerevisiae*, 80 mg) was then added to the contaminated cool wort, and fermentation was performed under static conditions at 20 °C for 7 days. The progress of the fermentation process was monitored daily by measuring the Brix levels (°Bx), which denote the sugar content in an aqueous solution. The cool wort was analyzed to confirm that no mycotoxin contamination was present before this set of fermentation experiments.

#### 3.4.2. Laboratory-Scale Wine Vinification

Red wine is prepared from must. In this study, to simplify the research method, frozen concentrated grape juice was used. This frozen concentrated juice was diluted with water to 19.5°Bx as must. Before use, the must was tested to confirm that it was not contaminated with mycotoxins. The must was autoclaved at 121 °C for 20 min and then cooled at room temperature. AFB_1_ was added to this must (350 mL) at a concentration of 10 ng·mL^−1^ (for determining its fate) or 10 μg·mL^−1^ (for differential analysis). Dried yeast (*S. cerevisiae*, 87.5 mg) was rehydrated and added to the contaminated must and fermented under static conditions at 25 °C for 7 days.

### 3.5. Sample Preparation

#### 3.5.1. Sample Preparation for Beer

Samples were obtained during the beer fermentation process and pretreated using an Oasis HLB solid phase cartridge (Waters, Milford, MA, USA). Each sample (1 mL) was added to 5% methanol:water (3 mL) and sonicated for 5 min. This solution was loaded on the HLB cartridge conditioned with 50% methanol (5 mL) and 5% methanol (5 mL), washed with 5% methanol (4 mL) 3 times, and then eluted with methanol (5 mL). The eluate was dried under a nitrogen atmosphere at 40 °C and the dried residue was dissolved with 10 mM ammonium acetate in water:acetonitrile = 85/15 solution (1 mL). The analytical solution was filtered through a PTFE filter with a 0.2-μm mesh and then transferred to a 2 mL glass vial. The mycotoxins present in the yeast crop required extraction with organic solvents before being cleaned up. Yeast crop (1 g) was added to 80% methanol (3 mL) and shaken vigorously for 10 min. The mixed solution was then centrifuged (3000 rpm, 5 min), and the supernatant was cleaned up using a solid phase column.

#### 3.5.2. Sample Preparation for Wine

The samples obtained during the wine fermentation process were pretreated using a published method for simultaneous mycotoxin analysis, with an Oasis HLB solid phase cartridge and a MultiSep #229 Ochra solid phase cartridge [20]. Briefly, a 5 mL sample was diluted with 25 mL of 10 mM ammonium acetate aqueous solution and was applied to an Oasis HLB cartridge. The cartridge was washed with 5 mL of 10 mM ammonium acetate aqueous solution and then eluted with 5 mL of 10 mM ammonium acetate aqueous solution:acetonitrile (1:1 *v*/*v*) and with 5 mL of acetonitrile. The eluate was dehydrated at 40 °C under a nitrogen stream. The dried sample was dissolved with 1 mL of water. Subsequently, 60 μL of formic acid and 5 mL of acetonitrile were added to the sample, and the mixture was applied to a MultiSep #229 Ochra cartridge. Four milliliters of the purified eluate was dehydrated at 40 °C under a nitrogen stream and was dissolved with 500 μL of 10 mM ammonium acetate aqueous solution:acetonitrile (85:15, *v*/*v*). Each sample was filtrated through a 0.20-μm PTFE filter immediately before UHPLC-MS/MS analysis. Extraction from wine lees was performed in the same way as that for the yeast crop of beer [3]. Wine lees (200 g) was added to 80% methanol (3 mL) and shaken vigorously for 10 min. The mixure was then centrifuged (3000 rpm, 5 min) and the supernatant was cleaned up using a solid phase cartridge as per the protocol used for wine fermentation.

### 3.6. Synthesis of Aflatoxin B_2a_

AFB_2a_ was synthesized with trifluoroacetic acid using the method reported by Takahashi *et al.* [17]. An AFB_1_ standard solution (3 μg·mL^−1^, 200 μL) was dried in a brown vial, and trifluoroacetic acid (200 μL) was added. Crude samples were kept under static conditions in a cool dark place for 1 h. This solution was then dried and resolved with acetonitrile (600 μL) to obtain an AFB_2a_ standard solution (1 μg·mL^−1^), which was then tested by LC-MSMS to confirm that all the AFB_1_ had been converted to AFB_2a_. 

### 3.7. Method Validation

The experimental method was statistically validated by comparing the RSD, recovery, and linearity (*r*). Repeatability and recovery were calculated from 5 experimental replicates per sample. Each noncontaminated sample was spiked with AFB_1_ at 5 ng·mL^−1^ before and after pretreatment. The linearity of the standard addition calibration curves was estimated from 8 data points: 0.5, 1, 2, 5, 10, 20, 50, and 100 ng·mL^−1^.

### 3.8. Analysis

The residual level of AFB_1_ in each sample was quantified using the standard addition calibration curve over a range from 0.5 to 100 ng·mL^−1^. The curve was based on samples that were prepared by spiking known amounts of analyte in the blank sample before pretreatment. The residual ratio was calculated from the concentration of each mycotoxin in the sample, with the initial concentration for the ground malt set at 100%. The following formula was used:

Residual ratio (%) = (concentration of AFB_1_ in sample)/(concentration of spiked AFB_1_) × 100 (1)

## 4. Conclusions

Here, we studied the fate of AFB_1_ during a laboratory-scale brewing and vinification process. The residual AFB_1_ levels were not greatly affected by either bottom or top fermentation in beer but were reduced during wine fermentation to 40% of their initial concentration. Differential analysis has previously shown that AFB_1_ is hydrated to the less toxic AFB_2a_ compound during the process of wine fermentation. Although both brewing and vinification involve fermentation by yeast, the residual ratios of AFB_1_ were significantly differed between these processes. This finding may be attributable to differences in the yeast strain, fermentation condition, or raw materials used. To our knowledge, this is the first study to show that the AFB_1_ concentration decreases during fermentation and that this is due to increased production of the hydrated derivative, AFB_2a_. These findings may assist in the development of strategies for reducing AFB_1_ contamination in alcoholic beverages.
